# Comparison of high- and low-dose radial extracorporeal shock wave therapy in carpal tunnel syndrome

**DOI:** 10.1590/1806-9282.20241815

**Published:** 2025-05-02

**Authors:** İsmail Ceylan, Büşra Kürtüncüoğlu, Figen Tuncay, Mehmet Canli, Halil Alkan, Abdulhamit Tayfur

**Affiliations:** 1 Kırşehir Ahi Evran University, School of Physical Therapy and Rehabilitation – Kırşehir, Turkey.; 2 Kırşehir Ahi Evran University, Faculty of Medicine, Department of Physical Medicine and Rehabilitation – Kırşehir, Turkey.; 3 Muş Alparslan University, Faculty of Health Sciences, Department of Physiotherapy and Rehabilitation – Muş, Turkey.

**Keywords:** Carpal tunnel syndrome, Extracorporeal shock wave therapy, Pain, Electromyography, Treatment

## Abstract

**OBJECTIVE:**

The aim of this study was to compare the efficacy of radial extracorporeal shock wave therapy administered at low vs. high pressures in patients with carpal tunnel syndrome.

**METHODS:**

Patients with carpal tunnel syndrome were randomized into two groups: low-dose group and high-dose group. Each patient underwent a total of four sessions of radial extracorporeal shock wave therapy, administered once a week. The radial extracorporeal shock wave therapy was delivered at 4.0 bars for the high-dose group and 1.5 bars for the low-dose group. Both groups received conventional physical therapy program consisting of transcutaneous electrical nerve stimulation, paraffin wax, orthoses, and tendon gliding exercises, three times per week over a 4-week duration. Outcome measures included pain levels, hand grip strength, pinch strength, the Boston Carpal Tunnel Syndrome Questionnaire, and nerve conduction studies.

**RESULTS:**

Both groups exhibited improvements across all measures, except for the nerve conduction studies parameters. In the intragroup analysis, statistically significant differences were observed with small-to-moderate effect sizes for median motor distal latency, median sensory nerve conduction velocity, median sensory distal latency, and the Boston functional status subscale, all favoring the high-dose group (p<0.05). In the low-dose group, a statistically significant difference with a moderate effect size was noted solely for hand grip strength (p<0.05).

**CONCLUSION:**

High-dose radial extracorporeal shock wave therapy was significantly better than low-dose radial extracorporeal shock wave therapy with small-to-moderate effect sizes in the recovery of the function and nerve conduction studies parameters of patients with carpal tunnel syndrome.

**Clinical Trials Registry:**

The study was registered on the Clinical Trials Registry (registration number: NCT05681663).

## INTRODUCTION

Carpal tunnel syndrome (CTS) is a prevalent form of neuropraxia affecting the upper extremity^1^. Non-steroidal anti-inflammatory drugs, local corticosteroid injections, diuretics, vitamin B supplements, rest splints, manual therapy techniques, and physical therapy modalities are utilized in the management of CTS^2^.

Extracorporeal shock wave therapy (ESWT) operates on the principle of directing high-amplitude sound waves toward the injured area of the body. Experimental studies indicated that ESWT enhanced nitric oxide production, repaired damaged axons, and promoted axonal regeneration^3,4^. Therefore, using the ESWT in patients with CTS could be a useful treatment option, especially for nerve healing.

In the literature, studies investigating the effect of radial ESWT (rESWT) on CTS used different pressure applications, and they reported that rESWT reduces the symptoms of CTS^5-7^. However, there is no consensus on which pressure value is more effective. Therefore, the aim of this study was to compare the effects of low (1.5 bars)- and high (4.0 bars)-pressure doses on CTS symptoms.

## METHODS

The study employed a randomized, single-blind, 1:1 parallel-group design and was conducted at Kırşehir Ahi Evran University from December 2022 to April 2023. Approval was granted by the local ethics committee on November 22, 2022 (2022-21/185). The study was conducted in accordance with the principles of the Declaration of Helsinki.

### Participants

Inclusion criteria consisted of individuals aged 18–65 years, experiencing paresthesia, pain, and vasomotor symptoms in the region associated with the median nerve for more than 6 weeks, a positive provocative test, and a neurophysiological assessment indicating mild-to-moderate median nerve lesion severity. Patients were excluded if they had undergone previous injections or surgery within the last 3 months, had severe CTS, exhibited any sensory and/or motor deficits in the ulnar or radial nerve, or had systemic conditions contributing to CTS.

### Randomization

**Figure 1:**
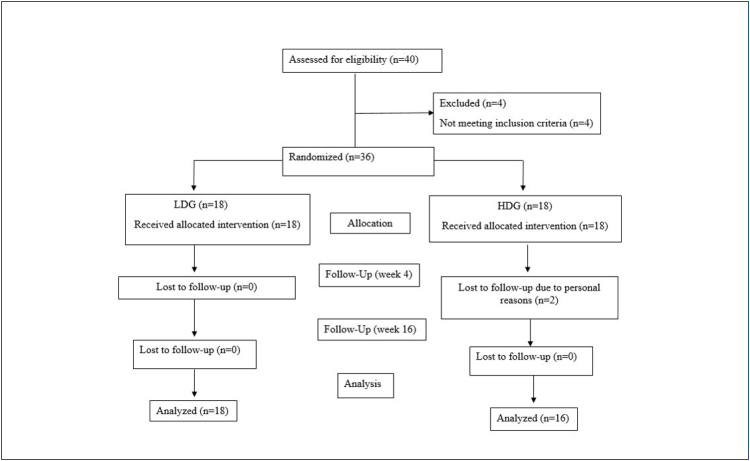
Study flowchart.

A randomization process was conducted to assign 36 patients with CTS to two study groups: high-dose group (HDG, n=18) and low-dose group (LDG, n=18), utilizing matched-pairs randomization based on age and sex (Figure 1).

### Blinding

All evaluations were conducted by the investigator, who remained blinded to the group assignments throughout the study. The patients and clinicians were not blinded.

### Interventions

The conventional physiotherapy program included transcutaneous electrical nerve stimulation (TENS), tendon gliding exercises, wrist resting splint, and paraffin wax treatment. This physiotherapy regimen was identical for both groups and was administered three times per week over a 4-week duration.

#### Transcutaneous electrical nerve stimulation

Conventional TENS was administered, with the pulse duration adjusted to 50–100 μs, and was delivered at a frequency of 100 Hz for a duration of 20 min, at an amplitude that did not induce muscle contractions^6^.

#### Tendon gliding exercises

The patients were directed to perform tendon gliding exercises as recommended by Totten and Hunter^8^.

#### Orthosis

The use of a night orthosis to keep the wrist in a neutral position was advised for each patient^5^.

#### Paraffin wax

Patients were asked to dip their hands up to their wrists in a paraffin cauldron. After each immersion, the patient's hand was removed from the paraffin cauldron, waited for 5 s, and dipped again. The patient's hand was covered with a bag and wrapped in a towel, and waited for 20 min^9^.

#### Radial extracorporeal shock wave therapy

Both the study groups received a total of four sessions of rESWT once a week. A Modus rESWT Touch Shock Waves device (Inceler Medikal, Ankara, Turkey) was used for rESWT with a frequency of 5 Hz and 2000 shock pulses for both groups^5^. The only difference was in pressures as the HDG received the rESWT with a pressure of 4.0 bars, while the LDG received 1.5 bars pressure ^5^.

### Outcome measurements

Outcome measures included pain intensity, hand grip strength, pinch strength, neurophysiological status, and functional status of the upper extremity. Measurements were taken before treatment (baseline), after treatment (week 4), and 12 weeks post-treatment (week 16). The nerve conduction studies (NCS) were conducted twice, at baseline and week 16.

### Primary outcomes

#### Visual analog scale

The patients were requested to indicate the intensity of the pain they experienced over the past 24 h by marking a 10-cm line scale, where 0="no pain" and 10="maximum pain." Pain was assessed using three distinct parameters: night pain, resting pain, and activity pain^10^.

#### Electroneuromyography

A Nihon Kohden Neuropack S1 MEB-9400 electroneuromyography (ENMG) device (Nihon Kohden Corp., Tokyo, Japan) was utilized. Values of median sensory distal latency (mSDL) <3.6 ms, median motor distal latency (mMDL) <4.2 ms, and median sensory nerve conduction velocity (mSNCV) >50 m/s were deemed normal^11^. The classification for CTS provided by the American Association of Neuromuscular and Electrodiagnostic Medicine (AANEM) was utilized^12^.

### Secondary outcomes

#### Hand grip strength

Grip strength for both hands was assessed using the Jamar® Hand Dynamometer (Patterson Medical, Warrenville, IL, USA), and the results were expressed in kilograms^13^.

#### Pinch strength

The pinch strength of both hands was measured using the Jamar® Pinch Meter (Patterson Medical, Warrenville, IL, USA), and the results were expressed in kilograms^14^.

#### Boston Carpal Tunnel Syndrome Questionnaire

The valid and reliable Turkish adaptation of the Boston Carpal Tunnel Syndrome Questionnaire (BCTSQ) was utilized, as it assesses both symptom severity and functional status in patients with CTS^15^.

### Sample size

The G-Power program (version 3.1.9.4, University of Düsseldorf, Düsseldorf, Germany) was utilized for this calculation. To achieve 80% power with an α error level of 0.05, repeated-measures analysis of variance (ANOVA) was conducted for both within-group and between-group interactions, employing a large effect size of 0.30 to account for the two groups.

### Statistical analysis

The IBM SPSS software (version 25.0, IBM Corp., NY, USA) was employed for statistical analysis. A two-way ANOVA was conducted to assess changes over time in the measured outcomes of the study groups and their group–time interactions. Eta squared (η²) was calculated to classify effect sizes as 0.02 (small), 0.13 (moderate), and 0.26 (large). A statistical significance level of p<0.05 was established.

## RESULTS

### Primary outcomes

There was a significant decrease in visual analog scale (VAS) rest, VAS activity, and VAS night measurements after treatment compared to pre-treatment measurements for both groups. While the highest decrease occurred in the VAS night of the HDG (7.06±2.89 vs. 2.13±2.78), the least decrease was in the VAS rest value of the LDG (5.11±2.17 vs. 2.61±2.12). The intragroup analyses of pain parameters indicated no significant group-by-time interaction (Table 1).

**Table 1:** Intergroup and intragroup analyses.

Variables	Groups	Baseline mean±SD	Week 4 mean±SD	Week 16 mean±SD	Intragroup p*	Intergroup p**	η^2^
VAS rest (cm)	LDG	5.11±2.17	3.28±1.87	2.61±2.12	**0.000** *	0.291	0.038
	HDG	5.5±3.18	4.25±3.38	2.38±2.87			
VAS activity (cm)	LDG	7.67±2.66	4.44±3.28	4.44±3.18	**0.000** *	0.076	0.077
	HDG	7.63±2.8	5.25±3.34	3.19±3.08			
VAS night (cm)	LDG	6.17±2.28	3.06±1.83	2.61±2.4	**0.000** *	0.248	0.043
	HDG	7.06±2.89	3.94±3.4	2.13±2.78			
mMDL (ms)	LDG	4.1±0.73	-	4.05±0.78	**0.014** *	**0.005** **	0.221
	HDG	4.35±1.22	-	3.6±0.78			
mSNCV (m/s)	LDG	41.75±6.74	-	40.22±9.07	0.084	**0.005** **	0.223
	HDG	39.86±8.42	-	45.75±10.05			
mSDL (ms)	LDG	2.99±0.6	-	3.3±1.51	0.784	**0.032** **	0.136
	HDG	3.11±0.74	-	2.71±0.64			
mSNAP	LDG	40.22±18.1	-	37.49±26.87	0.737	0.333	0.029
	HDG	20.31±11.38	-	25.9±10.81			
Grip strength (kg)	LDG	15.74±4.0	18.64±5.29	22.46±6.56	**0.000** *	**0.003** **	0.168
	HDG	20.16±6.03	22.61±6.25	22.99±5.73			
Pinch strength (kg)	LDG	5.25±1.79	6.28±1.9	6.21±1.93	**0.000** *	0.231	0.045
	HDG	6.4±1.57	6.87±1.66	7.09±1.51			
BCTSQ symptom severity	LDG	3.13±0.74	2.05±0.86	2.22±0.83	**0.000** *	0.162	0.055
	HDG	3.27±1.01	2.27±0.71	1.96±0.84			
BCTSQ functional status	LDG	3.25±0.89	2.78±0.95	2.87±0.91	**0.000** *	**0.04** **	0.103
	HDG	3.33±1.06	2.92±0.98	2.44±1.02			

SD: Standard deviation; LDG: low-dose group; HDG: high-dose group; mMDL: median motor distal latency; mSNCV: median sensory nerve conduction velocity; mSDL: median sensory distal latency; mSNAP: median sensory nerve action potential; BCTSQ: Boston Carpal Tunnel Syndrome Questionnaire; VAS: visual analog scale; kg: kilogram; cm: centimeter; ms: millisecond; m/s: meter/second; p<0.05;

* p: intragroup analysis;

** p: intergroup analysis;

η^2^: effect size,

statistically significant values are denoted in bold.

There was an improvement with moderate effect sizes in post-treatment (week 16) mMDL, mSNCV, mSDL, and median sensory nerve action potential (mSNAP) measurements compared to pre-treatment measurements for the HDG (Table 2).

**Table 2:** Sociodemographic and baseline clinical characteristics of the groups.

Variables		LDG (n=18) mean±SD	HDG (n=16) mean±SD	p*
Age (years)		51.89±12.21	56.88±7.25	0.164
Body mass index (kg/m^2^)		31.77±4.94	31.88±4.88	0.94
Sex (female:male)		15:3	15:1	0.347
CTS grade (mild:moderate)		3:15	4:12	0.549
Occupation (n)				
	Homemaker	11	15	0.164
	Teacher	4	1	
	Worker	3	0	
Affected side (right:left)		10:8	10:6	0.681
Dominant side (right:left)		18:0	16:0	-

SD: standard deviation; LDG: low-dose group; HDG: high-dose group; kg: kilogram; m: meter; CTS: carpal tunnel syndrome,

* p<0.05.

### Secondary outcomes

The sociodemographic information of the participants is shown in Table 2. There was a significant increase in grip and pinch strength measurements after treatment compared to pre-treatment measurements for both groups (Table 2). However, only grip strength indicated a statistically significant group-by-time interaction with a moderate effect size in favor of the LDG (HDG: 20.16±6.03 vs. 22.99±5.73; LDG: 15.74±4.0 vs. 22.46±6.56; p=0.003; η^2^=0.17).

According to the intergroup analysis, there was a significant decrease in BCTSQ symptom severity and functional status subscale measurements after treatment compared to pre-treatment measurements for both groups. However, only the BCTSQ functional status subscale (HDG: 3.33±1.06 vs. 2.44±1.02; LDG: 3.25±0.89 vs. 2.87±0.91; p=0.04; η^2^=0.10) indicated a significant group-by-time interaction with a small effect size in favor of the HDG (Table 2).

## DISCUSSION

In accordance with the findings, it was noted that both groups demonstrated improvements in pain, grip strength, pinch strength, and functional status following the intervention. However, those in the HDG were significantly better on the NCS parameters and BCTSQ functional status compared to those in the LDG 12 weeks after the intervention.

In a dose-dependent rESWT study, it was reported that three sessions of 4.0 bars rESWT improved mSNCV scores in patients with mild-to-moderate CTS^16^. In a randomized prospective study, the effectiveness of 1.5 bars rESWT and corticosteroid iontophoresis was compared in patients with CTS^17^. According to the literature, both low-dose and high-dose rESWT improved NCS parameters. Despite these findings, there were improvements in mMDL, mSNCV, mSDL and mSNAP measurements after the intervention in HDG.. Also, intragroup analysis of the mMDL, mSNCV, and mSDL measurements demonstrated a statistically significant intragroup interaction in favor of the HDG. In a systematic review on the use of NCS in the diagnosis of CTS, it was reported that the cutoff value for sensory latency was 3.37 ms (2.8–4 ms) and for motor latency was 4.28 ms (3.8–4.6 ms)^18^. When the values in our study were compared with the reported cutoff values, the sensory latency values in our study were significant in favor of the HDG (4.0 bars) and parallel to the literature.

There is presently no established standard treatment protocol regarding application frequency, energy intensity, or total shots for the use of rESWT in CTS^19^. However, numerous studies have employed two or more sessions of ESWT for chronic musculoskeletal disorders^5,17,19^. Consequently, clinical experience suggests that repeated sessions of ESWT may be more effective than a single application. In the present study, the more frequently used applications of rESWT in the literature were preferred, and rESWT was applied with 2,000 shots, 1.5 bars and 4.0 bars intensity of energy, and a frequency of 5 Hz. Especially, in the literature, there are conflicting applications for rESWT pressure intensity for treating CTS. A randomized controlled trial compared rESWT and corticosteroid iontophoresis, with an application of 1.5 bars rESWT^17^. In another study, rESWT, local corticosteroid injection, and splint groups were compared, with an application of 4.0 bars rESWT^5^. Thus, different pressure applications were used in studies examining the impact of rESWT treatment on CTS, and all studies reported that rESWT reduced the symptoms of CTS^5-7,17^. In our study, the effects of low- and high-pressure values on the symptoms of CTS were compared in order to investigate which pressure severity of rESWT is superior in the management of CTS. As a result, the application of rESWT at a high pressure (4.0 bars) in the treatment of CTS could be more effective in reducing clinical symptoms.

In this study, one of the limitations was that functional measurements were subjective based on patient statements. Also, the sample size was small, which may have limited the generalizability of the findings. Clinical functional tests could be useful as an objective method in future studies. Similarly, the use of ultrasound imaging which objectively measures the median nerve diameter would strengthen the level of evidence in future studies.

## CONCLUSION

Both groups experienced improvements in pain, grip strength, pinch strength, and functional status following the intervention. However, high-dose rESWT was significantly better than low-dose rESWT with small-to-moderate effect sizes in the recovery of the function and NCS parameters of patients with CTS.
